# Basic Disturbances of Information Processing in Psychosis Prediction

**DOI:** 10.3389/fpsyt.2013.00093

**Published:** 2013-08-23

**Authors:** Mitja Bodatsch, Joachim Klosterkötter, Ralf Müller, Stephan Ruhrmann

**Affiliations:** ^1^Department of Psychiatry and Psychotherapy, University of Cologne, Cologne, Germany

**Keywords:** basic symptoms, EEG/ERP, fMRI, prediction of psychosis, ultra-high risk

## Abstract

The basic symptoms (BS) approach provides a valid instrument in predicting psychosis onset and represents moreover a significant heuristic framework for research. The term “basic symptoms” denotes subtle changes of cognition and perception in the earliest and prodromal stages of psychosis development. BS are thought to correspond to disturbances of neural information processing. Following the heuristic implications of the BS approach, the present paper aims at exploring disturbances of information processing, revealed by functional magnetic resonance imaging (fMRI) and electro-encephalographic as characteristics of the at-risk state of psychosis. Furthermore, since high-risk studies employing ultra-high-risk criteria revealed non-conversion rates commonly exceeding 50%, thus warranting approaches that increase specificity, the potential contribution of neural information processing disturbances to psychosis prediction is reviewed. In summary, the at-risk state seems to be associated with information processing disturbances. Moreover, fMRI investigations suggested that disturbances of language processing domains might be a characteristic of the prodromal state. Neurophysiological studies revealed that disturbances of sensory processing may assist psychosis prediction in allowing for a quantification of risk in terms of magnitude and time. The latter finding represents a significant advancement since an estimation of the time to event has not yet been achieved by clinical approaches. Some evidence suggests a close relationship between self-experienced BS and neural information processing. With regard to future research, the relationship between neural information processing disturbances and different clinical risk concepts warrants further investigations. Thereby, a possible time sequence in the prodromal phase might be of particular interest.

## Introduction

Disturbances of information processing are a core feature of psychosis, particularly schizophrenia, with a significant impact on vulnerability and course (1, 2). Hence, with regard to the prediction of conversion to psychosis, indicators of such alterations are of special interest.

In the present review, we will consider three major areas of research, the basic disturbances concept and the event-related research employing either electro-/magnet-encephalographic (EEG/MEG), or functional magnetic resonance imaging (fMRI). Thereby results are emphasized that allow for a detection of those subjects clinically at high risk (3), who indeed developed a psychotic disorder.

“Basic symptoms” (BS) are conceptualized as a phenomenological counterpart of neural pathological changes in brain functioning and have been demonstrated to represent core features of psychotic disorders (4–8). These symptoms point to subtle, predominately only self-experienced disturbances in drive, affect, thinking, speech, perception, motor action, central vegetative functions, and stress tolerance, with full insight into their pathologic nature (8). The BS concept assumes that these subjective impairments are closely related to the pathophysiological aberrations underlying psychosis development (4, 6). Thus, the concept corresponds to the “subjective cognitive impairment” discussed as a risk indicator for dementia (9, 10). In their seminal psychopathological works on the BS concept, Süllwold and Huber (11) and Klosterkötter (12) conceived a three-domain model of the relationship between symptoms and pathophysiology changes, differentiating between a pre-phenomenal, a trans-phenomenal, and a phenomenal domain. The pre-phenomenal domain corresponds to the neurophysiological and neurochemical correlates of brain functioning. Aberrations in this domain lead to disturbances of neurocognitive processes, including de- and en-coding of information, gating, etc., and representing the trans-phenomenal domain. These disturbances are the source of the self-experienced BSs, which thus flag the transition from the trans-phenomenal to the phenomenal level and are understood as the basis for the further development of psychotic symptoms. The gradual transition toward full-blown positive symptoms interferes with the preexisting collective and individual anthropological information inventory that provides the patient’s cognitive scheme to explain aberrant cognitive-perceptive experiences (12). Thus, different to the BS, content and severity of positive symptoms are only distant, indirect reflections of the underlying pathophysiology, an assumption, which corresponds well to a recent model of psychosis development (13).

In line with the assumption of a close relationship to neurobiological changes, BS represent not only the earliest symptoms of psychosis development, but almost persistent phenomena that can be observed independently of positive and/or negative symptoms throughout large periods of the course of illness (5, 7, 14, 15).

Since BS are thought to mark the earliest stages of psychosis development, they have been employed to the aim of identifying subjects in presumably pre-psychotic stages of illness (7, 8, 14–16). The specificity of BS, which may be almost unspecific in the very early stages of psychosis development, has been thought to increase in the proximal pre-psychotic, legitimately called “prodromal” phase. In the yet largest study employing BSs to predict psychosis onset, 70% of the participants suffering from at least one BS developed schizophrenia within approximately 5 years, and 37% within the first 24 months of follow-up (14). Subsequent investigations have led to the establishment of two well-defined criteria, pointing either to a collection of highly predictive cognitive and perceptive disturbances (COPER) or to predominantly cognitive disturbances (COGDIS), respectively (7) (see Table 1 for an overview of the relevant cognitive and perceptive BSs). Subjects qualifying for the COPER criterion developed psychosis in 34.9% within 11 months on average (range 1–37, median 9 months) (17). The BS approach hence represents a valuable component in prediction research. However, the assessment of BS requires highly trained raters, particularly since insight and coping capability decline with the progression to full-blown psychosis (15). Furthermore, the valid evaluation of BS is often disabled by acute and/or prominent (attenuated) psychotic symptoms (15).

**Table 1:** **Predictive basic symptoms**.

Symptom	Description	Phenomenology
Thought interference	Intrusion of completely insignificant thoughts hindering concentration	“I can’t help thinking about other things, which is very distracting”
Thought perseveration	Obsessive like repetition of insignificant thoughts or mental images	“I always have to mull over what I just said. I can’t stop thinking about what I might have said wrong or what I could have added although I really don’t think that anything was wrong with what I said”
Thought pressure	Self-reported “chaos” of unrelated thoughts	“If I am stressed out my mind gets chaotic and I have great problems thinking straight. Too many thoughts come up at once”
Thought blockages	Sudden loss of the thread or train of thoughts	“Sometimes my thoughts just stop, are suddenly gone, like being cut off”
Disturbance of receptive language	Paralysis in the immediate comprehension of simple words/sentences, either read or heard	“I often can’t get the meaning of common words when I am reading”
Disturbance of expressive speech	Problems in producing appropriate words, sometimes experienced as a reduction in active vocabulary	“Sometimes I think it must appear as if English were really my second language, like I don’t know English very well because I have difficulties expressing myself. I forget the words”
Disturbances of abstract thinking	Inability to explain abstract contexts, sayings, or idioms	“Sometimes I get puzzled if a certain object or event only stands as a metaphor for some more general, abstract, or philosophical meaning”
Inability to divide attention	Interference of two non-demanding tasks on different sensual domains (e.g., verbal dialogue and motor action)	“Doing two things at once has become impossible even with the simplest things. I always have to concentrate on one thing at a time”
Captivation of attention	Involuntary captivation by details of the visual field that catches and holds the look.	“Sometimes an object really seems to stand out from the rest of what I see. My eyes then fix on it. It’s like being spellbound”
Decreased ability to discriminate between perception and ideas	The ability to allocate mental representations to their proper domain, e.g., to discriminate memory and fantasy	“I thought about my grandparents. Then a weird thing happened: I couldn’t remember if I knew my grandparents properly, if they were real, or if they were just in my imagination”
Unstable ideas of reference	Feelings of being directly addressed by a non-intentional environment with insight	“When I was listening to the radio the idea that the lyrics had some special meaning for me suddenly popped up into my head”
Derealization	Feelings of being detached from an “unreal” environment	“Sometimes, I feel disconnected from the world around me, like I’m under a glass cover”
Visual or acoustic perceptual disturbances	Perceptual observations, e.g., a wrong coloring, distorted shape, or changed sound quality/intensity with insight	“People suddenly seemed changed and had different hair colors”

*Adapted from Schultze-Lutter (15), with modifications. For COPER/COGDIS see Schultze-Lutter et al. (17)*.

The currently most widely used clinical criteria of psychosis prediction are the so called ultra-high-risk (UHR) symptoms (18). According to this approach, either attenuated psychotic symptoms (APSs) or brief, spontaneously remitting psychotic symptoms (BLIPSs) or a genetic liability in combination with an actual loss of functioning indicate a markedly increased risk for an imminent onset of full-blown psychosis (19). Although clinical studies during the recent decades have demonstrated that the prediction of psychosis is fairly possible this way, the respective research has simultaneously unearthed its main challenge: against the expectation, the majority of persons considered being in a pre-psychotic state does not develop full-blown psychosis in the foreseeable future (20). The clinical prediction of psychosis thus inherits significant uncertainty, as mirrored by non-conversion rates commonly exceeding 50% (at least within the available, almost too short observation periods) (21). Approaches that increase specificity are thus warranted.

Following the heuristic implications of the BS approach it can be assumed that the uncertainty of prediction results from an inherent ambiguity of (attenuated) psychotic symptoms. UHR criteria presumably represent disturbances of higher order integrative functions, reality testing, inner monitoring, and context evaluation possibly arising from various neural aberrations that do not necessarily comprise the specific processes leading to the development of, e.g., schizophrenia. The “prodrome,” however, is presumably characterized by specific disturbances of neural processing that are not mirrored by rather unspecific psychotic phenomena (11, 12, 21–24). In line with this, it could be hypothesized that in a population of persons clinically at-risk of developing psychosis, certain neural “markers” may assist in validly differentiating between future converters, i.e., persons displaying a truly prodromal state, and non-converters although these groups are clinically indistinguishable (25). In this regard, it appears not necessary that the hypothesized markers represent direct correlates of the observed clinical (psychotic) symptoms; it would be sufficient if their presence significantly increases the probability that the respective person converts to psychosis. From the view of the BS concept, disturbances of information processing seem to be the most promising candidates to this purpose since these have phenomenologically been demonstrated independent of psychotic symptoms (14, 15). Predictive BS display a low prevalence (particularly in comparison to subclinical psychotic symptoms) in the general population and in non-psychotic samples (26, 27). Moreover, even though some subtle, progressive structural changes might already occur in the pre-psychotic phase (28), functional disturbances may account best for the “fluid” dynamics of the at-risk state (22, 29). Objective measures provided by neuroimaging and neurophysiology, respectively, raise the opportunity to observe disturbances of information processing directly.

In the present paper, following the BS concept, we aim at exploring if and to what extent objective parameters of neural information processing allow for improving the prediction of psychosis onset. After an overview of the research on disturbances of neural information processing in the at-risk state, we will focus on findings indicating predictive capabilities.

## Methods

We used the following Medical Subject Heading (MeSH) categories: (fMRI OR EEG OR MEG) AND [UHR OR prodrome OR at-risk mental state (ARMS) OR clinical high risk (3)] AND (psychosis OR schizophrenia). Studies were screened for the employment of current risk criteria (COPER/COGDIS, UHR) and only studies based on clinical criteria were included. While not selecting for paradigms, inclusion was restricted to investigations employing fMRI, EEG, and MEG, respectively. The detailed review focused on studies comprising converters in the respective high-risk samples.

## Information Processing in Psychosis Development

### Functional neuroimaging

A broad body of literature has verified deficits associated with psychotic disorders by functional neuroimaging (fMRI). Particularly the functionality of the prefrontal cortex, contributing to executive and working memory functions, and moreover subserving emotion processing, reward, and social cognition, has been demonstrated to be impaired in schizophrenia (30).

#### High-risk state

Investigations employing tasks related to attention control, verbal fluency, and working memory, respectively, suggested almost consistently a gradual decline in frontal and striatal activation from the clinical risk state to chronic psychosis (30, 31). Further evidence suggests an impairment of fronto-temporal connectivity (30). Other studies demonstrated alterations in the neural correlates of emotion processing (32) and movement generation (33), respectively. Overall, the observed deficits tend to be significant compared to controls and tantamount, although less severe, compared to first-episode psychosis (30, 31).

A recent meta-analysis demonstrated a prefrontal dysfunction in the at-risk state across different paradigms (31). Additionally, reduced activation patterns have consistently been found in the anterior cingulate, the medial and superior frontal gyrus, and the inferior frontal gyrus, respectively (31). The anterior cingulate is involved in conflict monitoring, social cognition, and emotional processing (34, 35). The aforementioned areas of the frontal gyrus contribute to executive and memory functions (35). The inferior frontal gyrus has been demonstrated to be particularly involved in language processing with the observed deficits in at-risk subjects possibly relating to elevated dopamine in striatal regions (36, 37).

#### Prodromal state

Two studies yet compared fMRI measures in converters vs. non-converters (38–41). They focused on language processing and verbal fluency, respectively (39–41). Sabb and colleagues demonstrated a higher activation in the temporal lobes, the frontal operculum, the left precentral gyrus, the caudate, and striatal regions of future converters during the semantic logic condition of a language processing task (42). The authors reported a significant relationship between the left inferior frontal gyrus, the temporal lobe, the frontal gyrus, and psychopathological measures of thought disorder (42). The activation patterns in the anterior cingulate and the inferior frontal gyrus were inversely correlated with social adjustment scores at follow-up (42). Allen et al. demonstrated an increased activation in future converters, too, with regard to the left superior frontal gyrus, the middle frontal gyrus, parts of the brainstem, and the left hippocampus in a verbal fluency task (41). The authors additionally demonstrated an increased midbrain-prefrontal functional connectivity and an increased striatal dopamine metabolism in these subjects (41). Both studies, however, did not report any predictive models.

Taken together, functional disturbances in at-risk subjects have consistently been demonstrated, thereby pointing particularly to dysfunctions of frontal regions. However, definite conclusions should be deferred since the published fMRI studies vary markedly with regard to both, methodology and paradigms. Furthermore, the number of studies investigating differential deficits in converters and non-converters is yet sparse and none of the available studies reported discriminative statistics as required for predictor models.

### Neurophysiology

Contrary to the antecedent axiom that sensory brain regions simply relay neural representations of the environment to higher order networks, the very high complexity of early sensory information processing has to be appreciated: even at the earliest stages of sensory processing, incoming information is preconsciously filtered and digested via top-down and bottom-up loops (43). In focusing on sensory dysfunction as a potential etiological factor, neurophysiological research in the recent decades has revealed significant deficits in encephalographic correlates (EEG/MEG) of early information processing in schizophrenia. In this regard, the auditory system has been most extensively investigated, while sensory processing deficits are not restricted to this domain (43, 44). A recent investigation, for instance, demonstrated the intertwining of visual perception and higher order processing in a facial affect recognition task (45).

However, typical paradigms point to event-related potentials (42) appearing within 350 ms after stimulus presentation (43, 46–48). These ERPs are thought to straddle neural processing of increasing complexity from bottom pre-attentive filter functions to downstream sensory memory processing already involving attention control (43, 46–48).

#### High-risk state

Overall, sensory processing deficits in subjects displaying high-risk symptoms have been demonstrated to encompass the full range of processing, i.e., early as well as later stages. However, it is not yet clear how the observed disturbances differentially relate to the pathophysiology of psychosis development.

One of the earliest steps in neural processing contributes to filtering of sensory information. In at-risk subjects, significant deficits of the respective correlates have been demonstrated in studies on sensory gating (P50/N100 components) and prepulse inhibition (PPI) paradigms (47, 49–54). Although conceptualized as a pre-attentive measure, however, PPI might be moderated by selective attention (55). With regard to sensory gating, the majority of studies have found some though not all measures to be reduced in the clinical high-risk state, possibly tantamount to deficits observed in first-episode schizophrenia (47, 49–52). Neuroanatomically, temporoparietal, prefrontal, and hippocampal structures are thought to contribute to gating phenomena with the hippocampus regions CA3 being particularly involved in the later phases of stimulus processing (56, 57). Neurochemically, cholinergic neurotransmission via low-affinity nicotinic receptors and noradrenergic signaling through alpha-2-receptors has been found to particularly contribute to sensory gating (58).

Later stages of sensory information processing occupy the interface between perceptual and cognitive systems. A commonly used paradigm involves neural ability to discriminate deviant stimuli in a series of predictable standards (43, 46, 48). The mismatch negativity (MMN), which is thought to relate to context updating, may be significantly impaired in subjects at UHR compared to healthy controls (25, 50, 59–64). In comparison to first-episode psychosis, most studies demonstrated no statistically significant differences, and did neither find significant differences in comparison to recent onset psychosis, but a more pronounced deficit in at-risk subjects compared to chronic psychosis (25, 50, 59, 61, 62, 65). However, the number of future converters in the respective samples might significantly contribute to these statistical results and non-conversion may be associated with less severe or no MMN deficits (25, 59, 61, 63, 65). MMN deficits are currently best documented as potential markers of progression toward full-blown psychosis. Neural generators of the MMN have been localized bilaterally in the temporal cortex and in frontal regions with a predominance of the right hemisphere in tone paradigms and left sided generators in language paradigms (66). There may be two subcomponents of the MMN, the first generated in the superior temporal gyrus and the second in the inferior frontal gyrus, respectively (66, 67). The MMN seems to rely on glutamatergic and GABAergic neurotransmission, respectively, since the NMDA-receptor antagonist ketamine has repeatedly been shown to diminish MMN without affecting ERPs of similar latency and GABAergic substances have been demonstrated to attenuate MMN (58, 68, 69).

Investigations employing the P3 component, which is thought to reflect automatic processing of novelty and memory updating, have demonstrated significant deficits in at-risk subjects compared to healthy controls (44, 59, 62, 65, 70–76). Thereby, the P3 deficit in the at-risk state seems to be tantamount to the impairment observed in first-episode psychosis, but less severe compared to recent onset and chronic schizophrenia, respectively (44, 59, 62, 65, 72, 75). The P3 has been suggested as a potential marker of illness progression (73), but might more broadly indicate cognitive disturbances (58, 77). A recent investigation demonstrated a relationship between P3 and disturbances in receptive language in subjects displaying psychotic-like experiences (78). The P3 has been demonstrated to relate to frontal and posterior regions, particularly to precentral areas, insula, the parietal, and the inferior temporal cortex (56, 57). Since the P3 reflects higher order cognitive processes involved in attention and memory, it is sensitive to manipulations of various neurochemical pathways (58). Cholinergic stimulation, however, has been shown to rather specifically alter P3 (58).

#### Prodromal state

Nine studies have been identified that statistically compared neurophysiological measures in converters vs. non-converters (25, 47, 50, 52, 53, 61, 63, 70, 71, 73) (see Table 2 for overview).

**Table 2:** **Event-related potentials and prepulse inhibition in converters and non-converters**.

Study	Parameter	*N* at-risk (transitions)	Mean time to transition (months ± SD)	Predictive model reported	ARMS-T vs. ARMS-NT	Diagnoses after transition
Ziermans et al. (53)	PPI	42 (6)	Not reported	No	↓	Schizophrenia (*N* = 4), schizoaffective disorder (*N* = 1), bipolar disorder (*N* = 1)
Brockhaus-Dumke et al. (47)	Sensory gating	39 (21)	Not reported	No	↔	Schizophrenia (*N* = 20), schizoaffective disorder (*N* = 1)
Hsieh et al. (50)	Sensory gating MMN	67 (11)	Not reported	No	↔	Not reported
van Tricht et al. (52)	Sensory gating	61 (18)	Not reported	No	↓	Schizophrenia (*N* = 12), schizophreniform disorder (*N* = 3) schizoaffective disorder (*N* = 2), brief psychotic disorder (*N* = 1)
Bodatsch et al. (25)	MMN amplitude	62 (25)	7.0 ± 7.0	Yes	↓	Schizophrenia (*N* = 23), schizoaffective disorder (*N* = 1), delusional disorder (*N* = 1)
Shaikh et al. (63)	MMN amplitude	41 (10)	26.5 ± 26.6	No	↓	Schizophreniform disorder (*N* = 9), bipolar disorder (*N* = 1)
Higuchi et al. (61)	MMN amplitude	17 (4)	Not reported	No	↓	Schizophrenia
van Tricht et al. (73)	P300 amplitude	61 (18)	9.4 ± 7.2	Yes	↓	Not reported
Fusar-Poli et al. (70, 71)	P300 amplitude	39 (10)	Not reported	No	↔	Not reported

*PPI, prepulse inhibition; MMN, mismatch negativity; ARMS-T/-NT, at-risk mental state-transition/non-transition*.

*The arrows pointing downwards indicate a deficit in converters (independent of polarity of the respective ERP)*.

Ziermans and colleagues suggested a differential PPI deficit in converters and non-converters (53). Sensory gating measures were investigated by three studies (47, 50, 52). Thereof, two (47, 50) did not find significant differences between converters and non-converters. The P3 amplitude was demonstrated to be exclusively disturbed in future converters by one study (73), but to be unimpaired by another investigation (70, 71). Of the four published studies evaluating the MMN (25, 50, 61, 63), three consistently demonstrated a MMN deficit in future converters compared to non-converters (25, 61, 63, 79).

Predictive models employing neurophysiological parameters were provided by two published investigations (25, 73). Following a group of UHR subjects for 3 years, van Tricht and colleagues demonstrated that later converters to psychosis could be detected by deficits of the P§amplitude at baseline (73). Bodatsch et al. provided evidence that MMN amplitude deficits predict psychosis onset (25). This finding has recently been independently replicated (79). Based on a prognostic score derived from an MMN based Cox regression model, it was furthermore possible to generate two risk classes showing significantly different survival curves. Thus, it was possible to further stratify not only the risk for conversion (hazard rates 0.34 vs. 0.85) but also the mean times to event (20 vs. 13 months) (25).

Taken together, correlates of sensory processing and pending higher order functions indicate significant disturbances of neural information processing in at-risk subjects. Future research has to elucidate the pathophysiological meaning of these disturbances and their impact with regard to different outcomes of the at-risk state. However, first steps have been made to identify neural markers of psychosis development.

## Discussion

### Information processing disturbances in prodromal states of psychoses

Impairments of neural information processing in the pre-psychotic state have been demonstrated by a rising number of investigations, thereby providing evidence for the heuristic implications of the BS concept. However, although studies employing fMRI measures are sparse compared to investigations focusing on neurophysiology, significant evidence for information processing deficits is provided across methods and paradigms.

Functional magnetic resonance imaging studies most consistently demonstrated dysfunctions of regions differentially contributing to executive and memory functions, social cognition, emotional processing, and language processing, respectively (30, 31). Neurophysiological studies have provided large evidence for impairments of information processing, spanning from the earliest stages of sensory filtering up to memory and attention (25, 47, 49–54, 59–64, 72–76, 79).

However, since most studies did not comprise converters in their high-risk samples. As a considerable part of theses samples may not proceed to psychosis or even remit (at least clinically and temporally) from the at-risk state (23, 80, 81), the meaning of the respective findings is not yet clear. They may characterize early, subclinical stages of psychotic disorders or an increased vulnerability or transient changes (20, 22, 23).

### Predicting psychosis onset by information processing disturbances

Although fMRI investigations have demonstrated significant differences between converters and non-converters (41, 42), no predictive model has been reported in the respective publications. This, however, represents the litmus test for any potential indicator of an increased risk for developing a psychotic disorder.

Regarding neurophysiology, two studies yet established predictive models (25, 73) based on ERPs. Another approach integrating psychopathological and biological parameters in a one-step model has recently demonstrated increasing specificity of prediction (82).

However, since only one study investigated the P3 as a predictor of psychosis onset, this finding needs further corroboration.

Mismatch negativity amplitude reductions may predict conversion to psychosis and enable the stratification of risk into different classes (25). The respective classes have been demonstrated to differ significantly with regard to time to transition (25). Regarding sensory gating measures, progressive alterations may be associated with a prodromal development (52), but the observed deficits failed a correlation with the time until transition (52). However, sensory gating deficits seem to be moderated by the stage of illness since chronic schizophrenia patients exhibit more pronounced deficits compared to at-risk subjects (47, 50).

Sensory gating presumably refers to early pre-attentive processes, whereas P3 likely represents cognitive management of salient stimuli, and MMN may straddle bottom-up stimulus registration and top-down change detection. In synopsis of the currents literature, sensory gating deficits might predominantly indicate liability to psychotic experiences, P3 might be primarily susceptible to cognitive disturbances, and the MMN may be best suited to support the identification of future converters.

### Framing future research

The heuristic implications of the BS concept predict that disturbances of information processing can be observed largely independent of positive symptoms and are closely related to particular aberrations of brain functioning (6, 11, 12, 14, 15). However, although disparate with regard to the method, fMRI, neurophysiological measures, and BS psychopathology may thus converge on particular domains. Although recent investigations did not yet explicitly aim to elucidate this convergence, some results seem useful to generate future research hypotheses.

Among the most predictive BS, two concern disturbances of language processing (8, 14, 15). The left inferior frontal gyrus (IFG) is recruited in both, speech production and comprehension (83). fMRI investigations comparing converters and non-converters suggest differential disturbances located in the left IFG (31, 42). Correlations of IFG activity and increased striatal dopamine have been demonstrated in persons at-risk of psychosis (31, 37), and normalization of IFG activity has been associated with a favorable course of the risk syndrome (39). In turn, Sabb and colleagues demonstrated an inverse relationship between IFG activity and future functioning (42). Furthermore, neural generators of the MMN have been located in the IFG (66). The P3 might be related to domains involved in language processing as well (78). At least, an intertwining of the P3 with dysfunctions of language comprehension has been found in subjects displaying psychotic-like experiences (78). Taken together, it can be hypothesized that the language related BS may be mirrored by particular EEG disturbances that relate to language processing domains, which have been found aberrantly activated in fMRI investigations. Apart from that, the evidence for a close relationship between BS phenomenology and particular neural activity appears yet less univocal. However, deficits in neurophysiological parameters seem to be more closely related to cognitive deficits than to psychotic symptoms (77). Sensory gating measures, for instance, have been found to correlate with impairments in sustained attention (77), although this finding is not uncontested, and attention disturbances have been identified as predictive symptoms in prospective studies employing BS criteria (8, 14, 15). In turn, correlations of gating measures to positive or negative symptoms have been denied by the majority of studies (77).

The relationship between BS and particular brain regions has still to be investigated directly. However, the synopsis of the current literature provides an impetus for future research.

### Can neurobiological variables enhance prediction of psychosis?

Since all samples discussed in this review are preselected by clinical risk criteria, it remains unclear if objective measures would perform equally well as screening tools in non-selected, help-seeking samples. Furthermore, their predictive value beyond clinical criteria has still to be evaluated. However, different to any clinical approach, neurophysiological parameters provide an estimate of the remaining time until transition (25, 79), which is most important for targeted intervention. Another aspect relates to reliability. The clinical assessment of at-risk states needs highly trained specialists to overcome the clinical ambiguity of certain symptoms (80). Although technically not trivial, the objectivity of parameters of information processing might hence provide decisive advantage.

The specificity of neurobiological parameters represents another major topic. Neural information processing deficits have been observed in non-psychotic disorders, too, as well as in healthy individuals, although some parameters may be more specific to psychosis (43, 84–87). However, it appears debatable if any non-clinical approach should actually be intended to identify high-risk subjects irrespective of clinical criteria, e.g., in population samples. Currently, the best approach may be a two-step algorithm of risk detection and subsequent risk stratification (22). The integration of multiple different measures in a single step may increase specificity (82) but will likely result in an undesirably loss of sensitivity (22). A sequential algorithm of risk estimation would avoid this disadvantage by employing firstly measures with a high sensitivity (clinical risk criteria) and subsequently predictors with a presumably high specificity (e.g., parameters of information processing) (22). Such an approach may be best suited not only to validly identify high-risk subjects and enrich samples for research purposes, but also to enable stratification of risk and with that individualized risk estimation as a major step toward needs-adapted prevention.

## Conclusion

The heuristic implications of the basic disturbances concept predict that particularly aberrations of neural information processing represent the objective counterparts of cognitive and perceptive BS. The current literature provides evidence that information processing deficits can discriminate at-risk subjects converting to psychosis from those who will not develop a psychotic disorder. In fMRI investigations, group differences have been demonstrated particularly with regard to brain regions involved in language processing. Event-related potentials may enable a quantification of risk in terms of magnitude and time. Particularly the latter finding represents a significant advancement compared to clinical approaches. In synopsis of the literature, some findings seem to support the assumed close relationship between self-experienced BS and neural information processing. Disturbances of language function have been identified as predictive symptoms in clinical BS studies and neural correlates have been demonstrated in both, fMRI and neurophysiological investigations. Even though the relationship between BS phenomenology and neural activity appears yet largely unknown in general, deficits in neurophysiological parameters seem to be more closely related to cognitive deficits than to psychotic symptoms.

Taken together, the BS concept provides not only a valid instrument in predicting psychosis onset but represents moreover a significant heuristic framework for future research.

## Conflict of Interest Statement

The authors declare that the research was conducted in the absence of any commercial or financial relationships that could be construed as a potential conflict of interest.
